# Supplementary material for: The Polycomb group protein CBX6 is an essential regulator of embryonic stem cell identity
Source: Nat Commun. 2017 Nov 1;8:1235. doi: 10.1038/s41467-017-01464-w (PMC5663739; doi:10.1038/s41467-017-01464-w)
Supplement: Supplementary file 1 — Supplementary Information [file 41467_2017_1464_MOESM1_ESM.pdf]

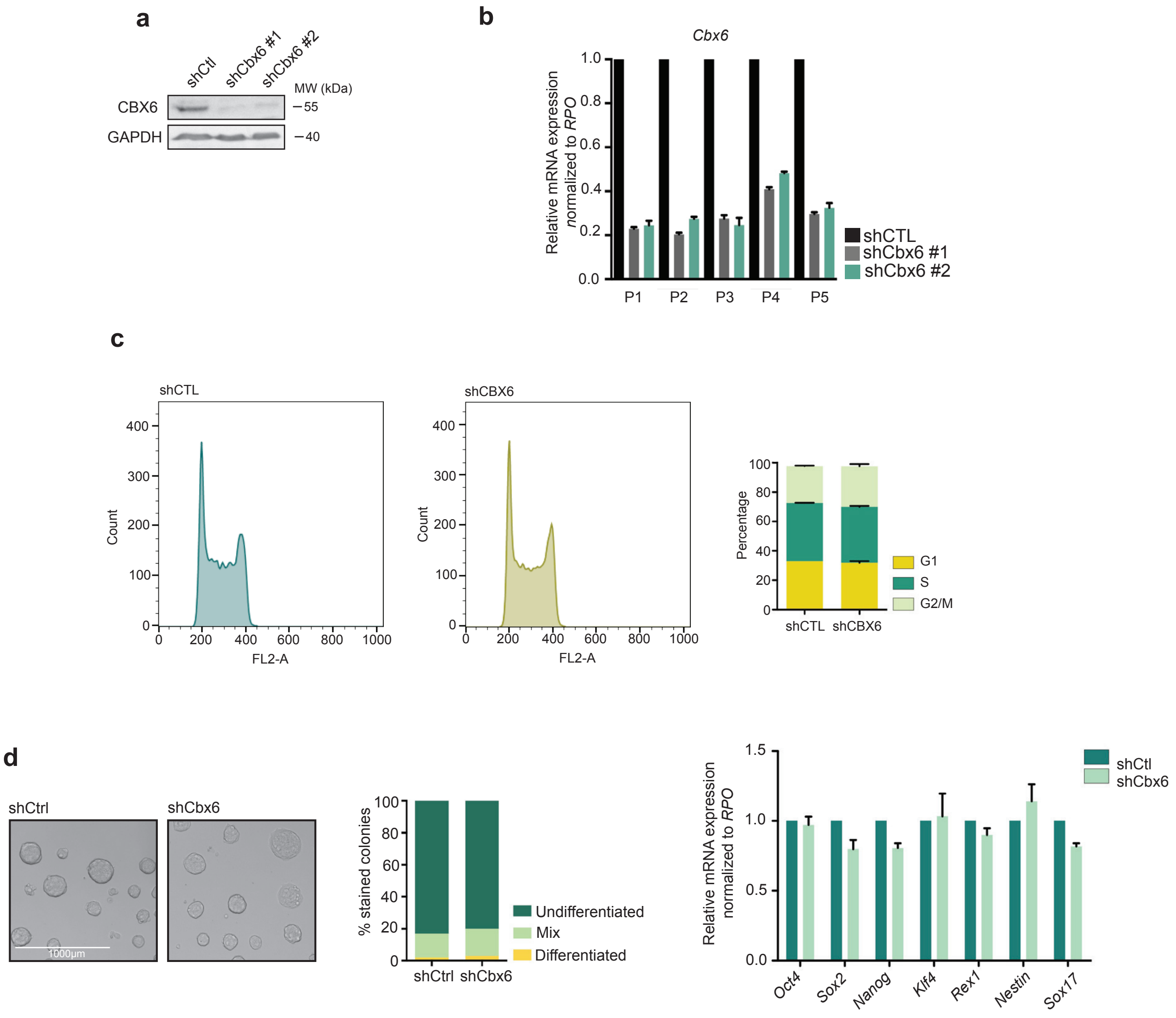

Supplementary Figure 1. (**a**) Western blot analysis of CBX6 in control (shCtl) and CBX6-depleted (shCbx6#1 and #2) ESCs. GAPDH was used as a protein-loading control. (**b**) RT-qPCR analysis of CBX6 in control and CBX6-depleted ESCs. Results are shown relative to shCtl and normalized to *Rpo*. Error bars represent SD of two independent experiments (P, passage). (**c**) BrdU staining in control (shCtl) and CBX6-depleted (shCbx6). (**d**) (left panel) Phase contrast image of shCtl and shCbx6 ESC lines. (middle panel) Quantification of the AP staining assays, representing the mean of two independent experiments in which around 40 random colonies were counted. (right panel) RT-qPCR analysis of control and CBX6-depleted ESCs. Results are shown relative to shCtl and are normalized to the housekeeping gene *Rpo*. Error bars represent standard deviation (SD) of two independent experiments.

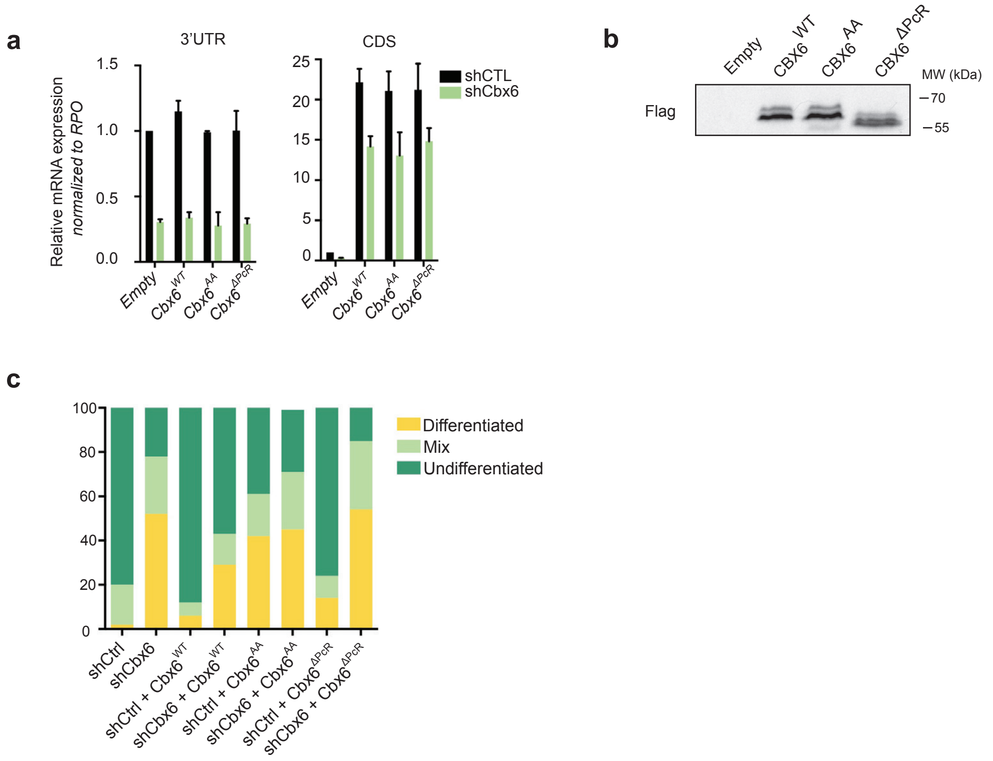

Supplementary Figure 2. (**a**) Western blot analysis of overexpressing CBX6-Flag constructs. (**b**) RT-qPCR mRNA expression analysis of endogenous CBX6 (3′-UTR) or total CBX6 (CDS, coding sequence) in control (shCtl) and CBX6-depleted ESCs (shCBX6) overexpressing $CBX6^{WT}$, $CBX6^{AA}$ or $CBX6^{\Delta PcR}$!'br an empty vector. Results are shown relative to shCtl, and normalized to *Rpo*. Error bars represent SD of two independent experiments."(**c**) Quantification of the AP staining assays, representing the mean of two independent experiments in which about 40 random colonies were counted.

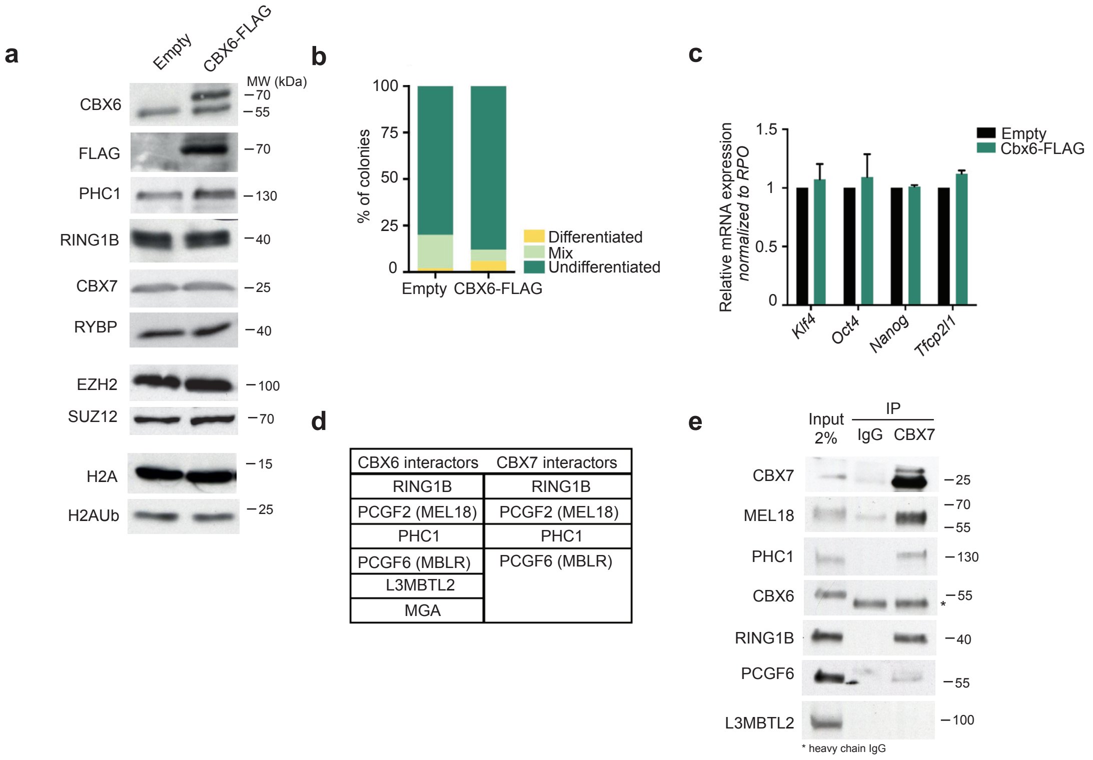

Supplementary Figure 3. (**a**) Western blot analysis of PRC1 subunits (PHC1, RING1B, MEL18, CBX7 and RYBP), PRC2 (EZH2 and SUZ12) and H2A/H2AUb, in control (empty) and CBX6-FLAG overexpressing cell lines. (**b**) Quantification of the AP staining assays, representing the mean of three independent experiments in which around 100 random colonies were counted. (**c**) RT-qPCR analysis of control and CBX6-depleted ESCs. Results are shown relative to shCtl and normalized to the housekeeping gene *Rpo*. Error bars represent standard deviation (SD) of two independent experiments. (**d**) Comparative table between CBX6 and CBX7 interactors. CBX7 information was extracted from Tavares et al.[9] (**e**) Co-IPs from total ESC extracts using an antibody against CBX7. Western blots of different proteins are shown.

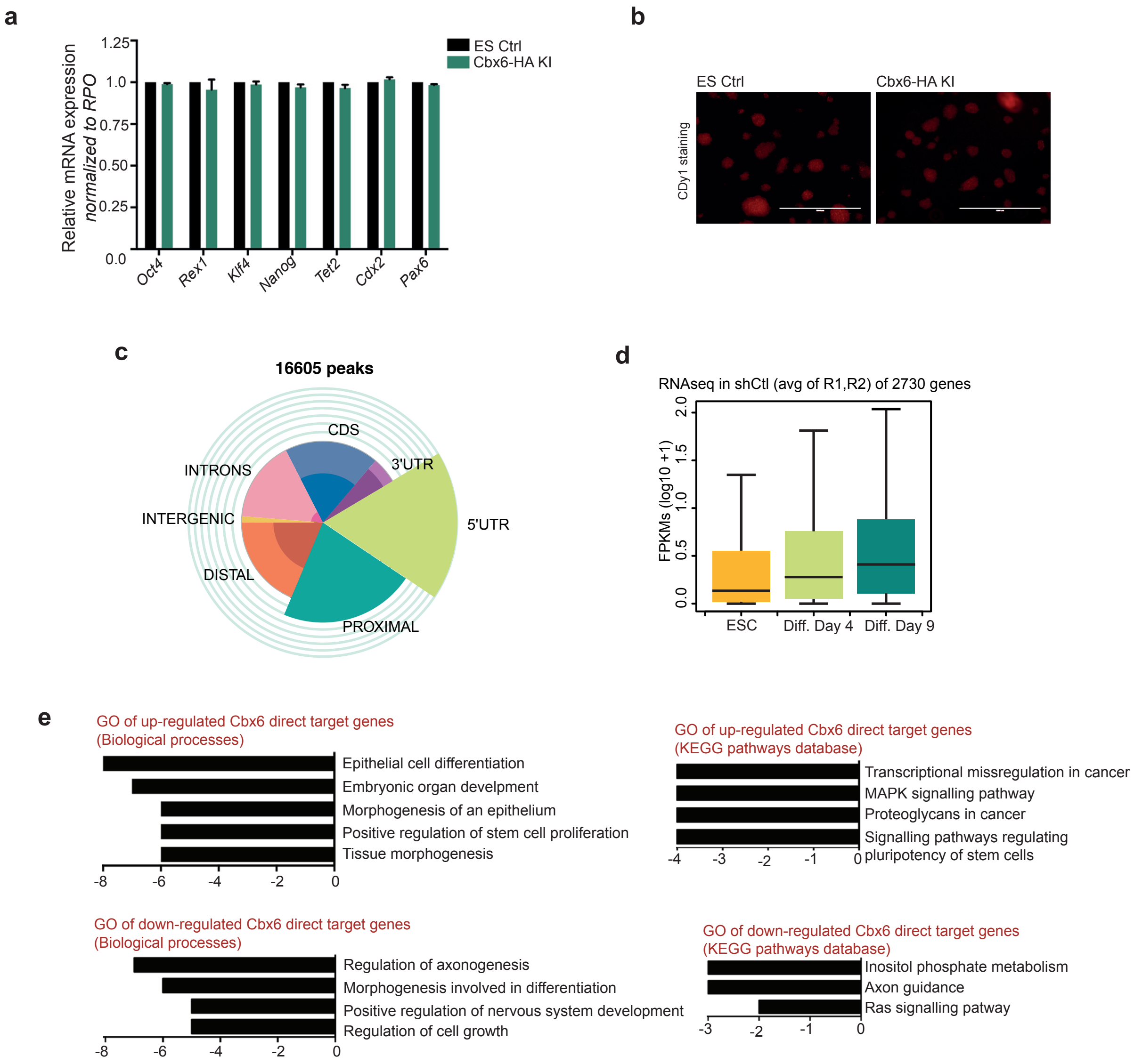

Supplementary Figure 4. (**a**) qRT-PCR analysis of control and CBX6-3×HA knockin ESCs. Results are shown relative to control and normalized to Rpo. Error bars represent SD of two independent experiments. (**b**) CDy1 staining of controlCBX6-HA knockin ESCs. (**c**) Genomic distribution of ChIPseq peaks of Cbx6 compared to the whole genome. Spie chart that superimposes two different pie charts. The base pie chart represents the distribution of Cbx6 peaks fitting on each class of genomic region (background image). The second pie chart displays the same categories with the radius corrected by the genome-wide distribution of each gene feature (foreground image). DISTAL region is the region within 2.5 Kbp and 0.5 Kbp upstream of the TSS. PROXIMAL region is the region within 0.5 Kbp and the TSS. UTR is UnTRanslated sequence. CDS is the protein CoDing Sequence. INTRONS are intronic regions. INTERGENIC is the rest of the genome. TSS is the Transcription Start Site (**d**) UCSC genome browser representation of CBX6 ChIP-seq signal at two representative genomic regions from ESCs expressing an empty vector or CBX6-HA. (**e**) GO analysis of genes deregulated following CBX6 knockdown; *P* values are plotted in –log.

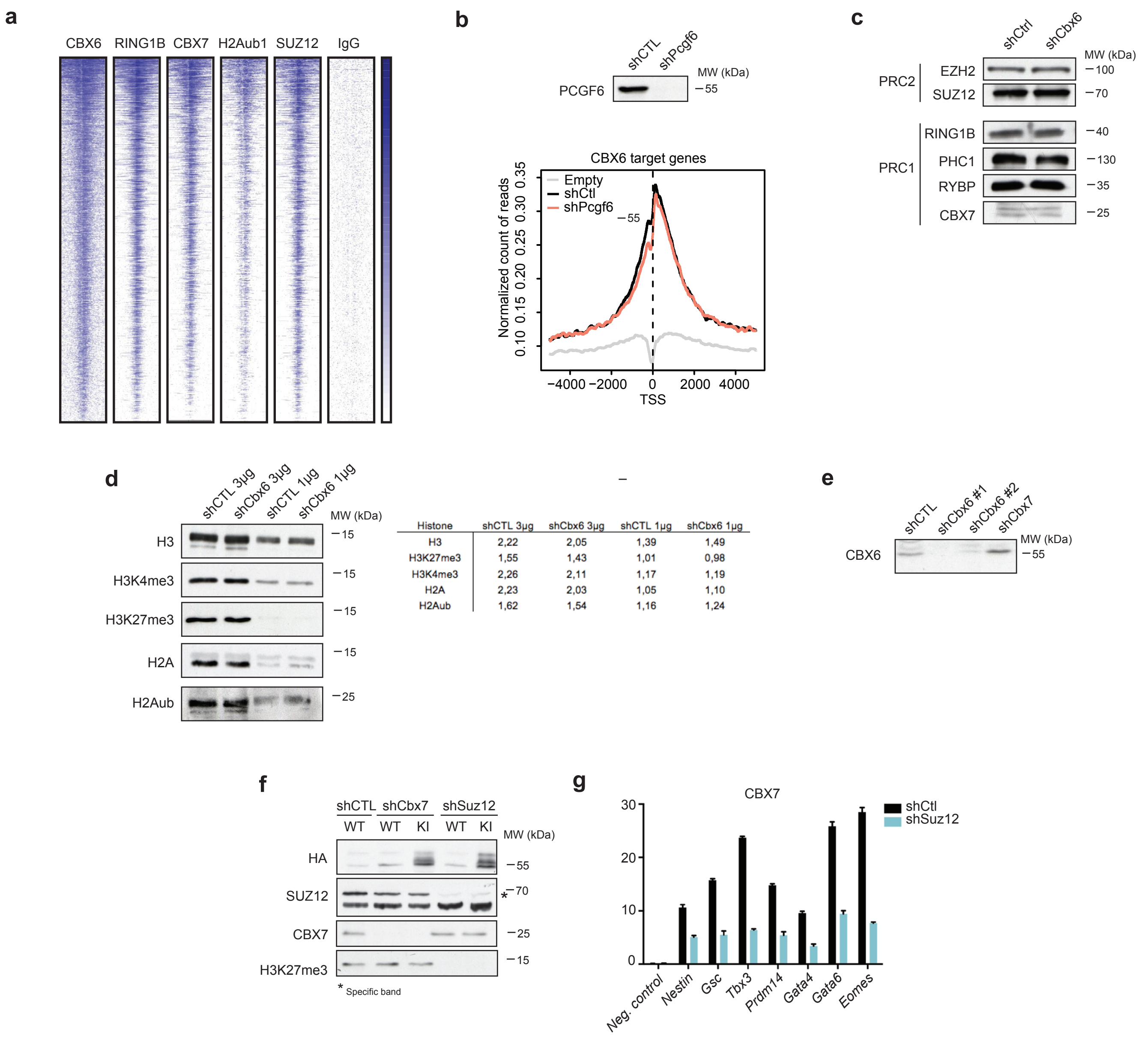

Histone	shCTL 3µg	shCbx6 3µg	shCTL 1µg	shCbx6 1µg
H3	2,22	2,05	1,39	1,49
H3K27me3	1,55	1,43	1,01	0,98
H3K4me3	2,26	2,11	1,17	1,19
H2A	2,23	2,03	1,05	1,10
H2Aub	1,62	1,54	1,16	1,24

Supplementary Figure 5. (**a**) ChIPseq heatmaps of CBX6, RING1B, CBX7, H2AK119ub, SUZ12 and IgG. Target genes were defined by the presence of one or more CBX6 peak within ±2.5 kb of a given transcriptional start site (TSS). (**b**) (up) Western blot analysis of PCGF6 in shCtl and shPcgf6 ESCs. (down) TSS (+/– 5 kb) enrichment plot of CBX6 ChIP-seq in shCtl and shPcgf6 ESCs. (**c**) Western blot analysis of PRC2 (EZH2 and SUZ12) and PRC1 (RING1B, PHC1, RYBP, CBX7 and PCGF2) from either shCtl or shCBX6 ESCs. (**d**) Western blot analysis and quantification of histone modifications in shCtl and shCbx6 ESCs. (**e**) Western blot analysis of CBX6 in shCtl, shCbx6 and shCbx7 in ESCs (**f**) Western blot analysis of CBX6, SUZ12, CBX7 and H3K27me3 in shCtl and shSuz12 ESCs. (**g**) ChIP-qPCR of CBX7 in shCTL and shSuz12 ESCs. An intergenic region was used as a negative control gene. Results are shown relative to percentage of input. Error bars represent SD of two biological replicates

Figure 3c

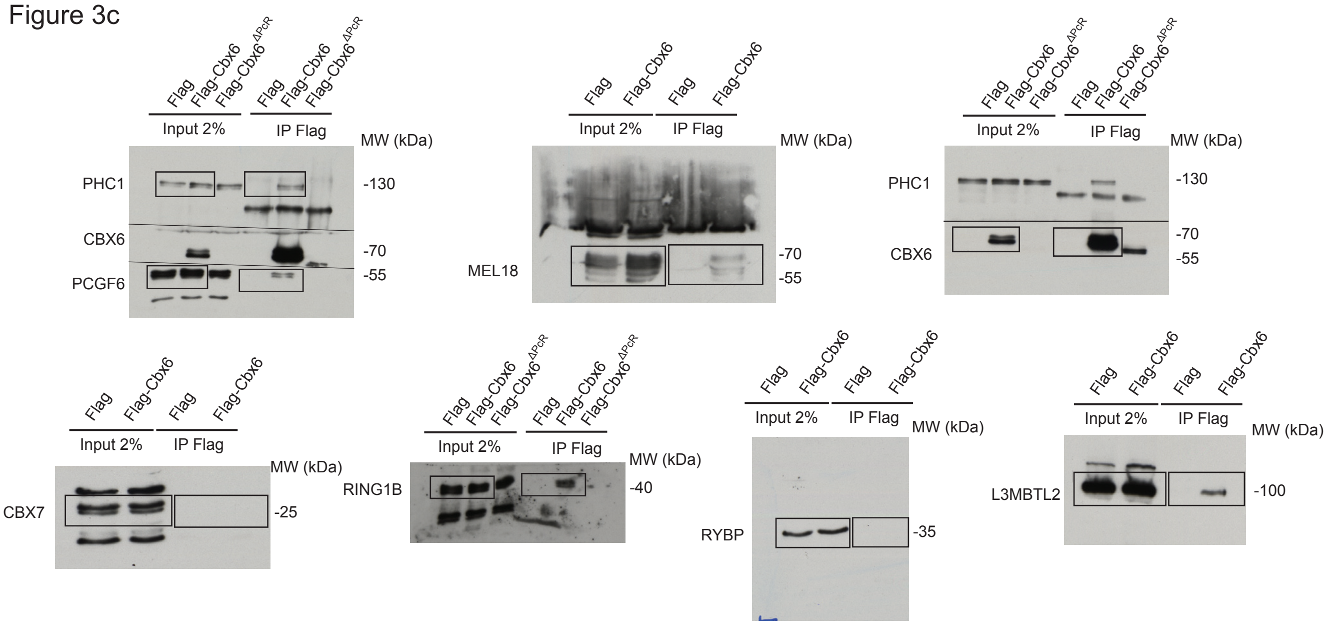

Figure 3d

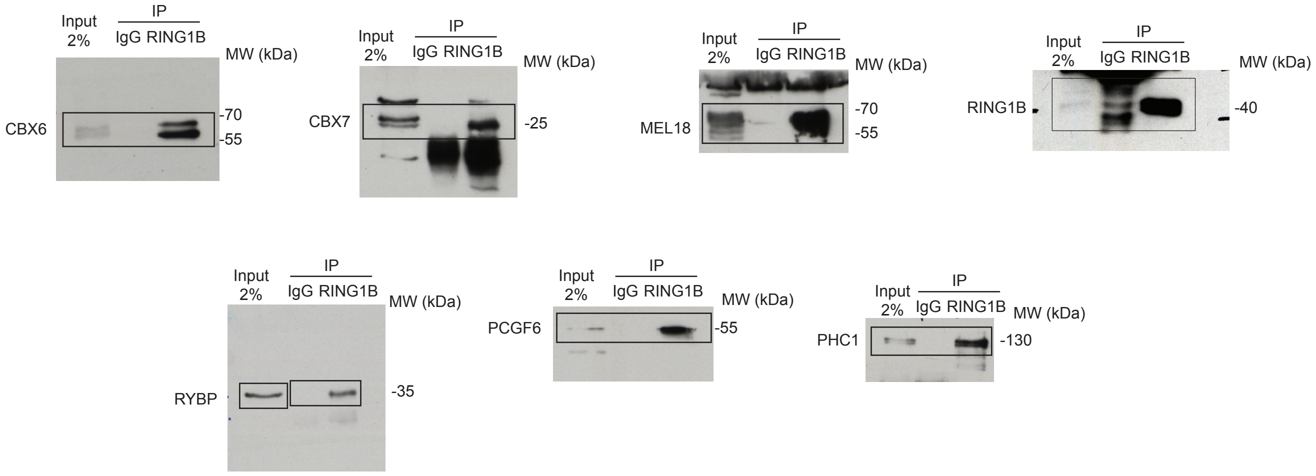

Supplementary Figure 6. Uncropped western blots related to Figure 3.

Antibodies	Specie	Provider	Reference	ChIP	IP	WB
Cbx6	Rabbit pAb	In house				1/1000
Cbx7	Rabbit pAb	Abcam	ab21873	5ug		1/500
Ezh2	Mouse mAb	In house				1/2000
Flag	Mouse mAb	Sigma	A2220		5ug	1/1000
Gapdh	Mouse mAb	Santa Cruz	SC-32233			1/5000
HA	Rabbit pAb	BioLegend	902301	5ul		1/2000
HA	Mouse mAb	In house				1/100
H2A	Rabbit pAb	Abcam	ab18255	3ul		1/5000
H2AK119Ub	Rabbit pAb	Cell Signaling	8240s	5ul		1/5000
H3	Rabbit pAb	Abcam	ab1791	2ug		1/5000
IgG	Rabbit pAb	Abcam	ab46540	5ug	5ug	
Mel18	Rabbit pAb	Santa Cruz	sc 10744	5ug	5ug	1/2000
Pcgf6	Rabbit pAb	Dr. Pasini lab		5ug	2ug	1/1500
Phc1	Mouse mAb	Active motif	39723	5ug	5ug	1/2000
Ring1B	Rabbit pAb	In house		8ul		1/2000
Ring1B	Mouse mAb	MBL	D139-3		5ug	
Rybp	Rabbit pAb	Millipore	3637	5ug	5ug	1/2000
Suz12	Rabbit pAb	Abcam	ab12073-100	5ug		1/2000
L3mbtl2	Rabbit pAb	Sigma	HPA000815			1/100

Supplementary Table 1. List of antibodies used and their application.

Gene		Primers 5'-3'
ChIP		
EOMES	Fw	GGCGCAGGGAATCTTAACTG
	Rv	AAGACCCAACATGAGCCTGA
Gata4	Fw	TCTCCAGCACCCATCAGTTT
	Rv	TTCTGGGAAACTGGAGCTGG
Gata6	Fw	TCTTCCTGCTCTCCCCTTTG
	Rv	CACAAACCGCCTTACAACCA
Gsc	Fw	GCCAGGTGAGTAAAGCAAGC
	Rv	GCCTGGGCTACAACAGCTAC
Nestin	Fw	CTCGGGAGAGTCGCTTAGAG
	Rv	GCTTGGTTTTACCAGGGACA
Prdm14	Fw	GTGTCACCCGACTGAAAGGT
	Rv	GTTCGTTTTGTTGGGCTGAT
Tbx3	Fw	GGCTGACTGTTGACGTTTGA
	Rv	CGGTCTCCTTCAATGGAAAA
mRNA		
Cbx6	Fw	GCCGAATCCATCATTAAACG
	Rv	TTGGGTTTAGGTCCCCTCTT
Cbx7	Fw	AGCCTCGGGGTATAGGAAGA
	Rv	CGGTGATGTCAGTCACGGTA
Esrrb	Fw	GGCGTTCTTCAAGAGAACCA
	Rv	TCCGTTTGGTGATCTCACAT
Klf4	Fw	CAGCCATGTCAGACTCGCC
	Rv	GTTTTTAATCTTCGTTGACTTTGGG
Nanog	Fw	AGGCTGATTTGGTTGGTGTC
	Rv	CCAGGAAGACCCACACTCAT
Nestin	Fw	TCCCCTGAGGACCAGGAGT
	Rv	GTCTCAGGACAGTGCTGAGCCTTC
Nr0b1	Fw	TCCAGGCCATCAAGAGTTTC
	Rv	ATCTGCTGGGTTCTCCACTG
Oct4	Fw	GAGGAGTCCCAGGACATGAA
	Rv	AGATGGTGGTCTGGCTGAAC
Rest	Fw	GTGCGAACTCACACAGGAGA
	Rv	AAGAGGTTTAGGCCCGTTGT
Rpo	Fw	TTCATTGTGGGAGCAGAC
	Rv	CAGCAGTTTCTCCAGAGC
Sox2	Fw	CTGCAGTACAACTCCATGACCAG
	Rv	GGACTTGACCACAGAGCCCAT
Tcfcp2l1	Fw	AGCATCATCCGTGTCGTTTT
	Rv	CAGTGCACCTGAATGAATGC
Tet2	Fw	GCCAGAAGCAAGAAACCAAG
	Rv	CCTTCCTTCAGACCCAAACA

Supplementary Table 2. Mouse primers used for mRNA and ChIP analysis.
